# Molecular classification and association with survival outcomes in high-intermediate and high-risk early-stage endometrial cancers: Ancillary analysis of GOG-0249☆

**DOI:** 10.1016/j.ygyno.2026.05.013

**Published:** 2026-05-26

**Authors:** Lindsay M. Kuroki, Danielle M. Enserro, Heather A. Lankes, Kyle C. Strickland, Rebecca Previs, Aine Clements, Matthew A. Powell, Ann Klopp, Elena Ratner, Paul DiSilvestro, Casey Cosgrove, Guilherme Henrique Cantuaria, Nicola M. Spirtos, Michael Carney, Mitchell Edelson, Gottfried Konecny, Penny Anderson, Christopher Tarney, Britt K. Erickson, Meredith Newton, Krishnansu S. Tewari, Premal H. Thaker, David Warshal, Amy Armstrong, Xun Clare Zhou, Angeles Alvarez Secord

**Affiliations:** a Washington University School of Medicine, Siteman Cancer Center, St. Louis, MO, United States of America; b NRG Statistical Center, Roswell Park Comprehensive Cancer Center, Buffalo, NY, United States of America; c NRG Oncology Operations Center-Philadelphia East, Philadelphia and The Ohio State University Wexner Medical Center, Columbus, OH, United States of America; d Labcorp Oncology, Durham, NC, United States of America; e Duke University Medical Center, Duke Cancer Institute, Durham, NC, United States of America; f OhioHealth Physician Group in Columbus, OH, United States of America; g MD Anderson Cancer Center, Houston, TX, United States of America; h Yale Cancer Center/Smilow Cancer Hospital, New Haven, CT, United States of America; i Women & Infants Hospital, Providence, RI, United States of America; j The Ohio State University Wexner Medical Center and James Cancer Hospital, Columbus, OH, United States of America; k Georgia Center for Oncology Research and Education, Northside Hospital Cancer Institute, Atlanta, GA, United States of America; l Women's Cancer Center of Nevada, Las Vegas, NV, United States of America; m John A. Burns School of Medicine, University of Hawaii Cancer Center, Honolulu, HI, United States of America; n Jefferson Abington Hospital, Sidney Kimmel Cancer Center, Thomas Jefferson University, Philadelphia, PA, United States of America; o UCLA Jonsson Comprehensive Cancer Center, Los Angeles, CA, United States of America; p Fox Chase Cancer Center, Philadelphia, PA, United States of America; q Uniformed Services University of the Health Sciences, Walter Reed National Military Medical Center, Bethesda, MD, United States of America; r University of Minnesota, Minneapolis, MN, United States of America; s University of North Carolina School of Medicine, Chapel Hill, NC, United States of America; t University of California, Irvine, CA, United States of America; u Cooper Hospital/University Medical Center, Camden, NJ, United States of America; v University Hospitals Cleveland Medical Center, Case Comprehensive Cancer Center, Cleveland, OH, United States of America; w Hartford HealthCare, Hartford, CT, United States of America

**Keywords:** Endometrial cancer, Molecular classification, Survival

## Abstract

**Objective.:**

Determine whether mismatch repair (MMR) and p53 expression predict recurrence-free survival (RFS) and overall survival (OS) in early-stage endometrial cancer (EC) patients treated with vaginal cuff brachytherapy plus chemotherapy (VCB/C) versus pelvic radiation therapy (RT).

**Methods.:**

In the GOG-0249 trial, 601 patients with high-intermediate risk (HIR) early-stage EC were randomized to VCB/C (carboplatin/paclitaxel for 3 cycles) or pelvic RT. Molecular subgroups, deficient MMR (dMMR), p53 wildtype (p53wt), p53 abnormal (p53abn), were determined by immunohistochemistry. Five-year RFS and OS were analyzed using Kaplan-Meier curves and Cox proportional hazards models, adjusting for lymphadenectomy, planned VCB, and treatment group.

**Results.:**

Among 315 patients, 23.5% had EC exhibiting p53abn expression, 32.1% had dMMR, and 44.4% had p53wt. p53abn EC was associated with older age (*p* = 0.004), serous histology (*p* < 0.001), lymphadenectomy (*p* = 0.02), and planned VCB (p < 0.001). Five-year RFS and OS were worse in patients with p53abn cancers, 58.7% [Hazard Ratio (HR) = 4.0 (95% Confidence Interval (CI): 2.1–7.5)] and 70.7% [HR = 9.4 (95%CI: 3.8–23.6)] and dMMR cancers 74.4% [HR = 1.8 (95%CI: 0.9–3.3)] and 84.3% [HR = 3.0 (95%CI: 1.2–7.8)] compared to those with p53wt (referent) (83.4% and 95.3%) (p < 0.001). The RFS (*p* = 0.7) and OS (*p* = 0.8) rates did not differ by treatment within molecular subgroups.

**Conclusion.:**

Molecular classification is prognostic for survival outcomes in patients with stage I-II HIR/high-risk EC. Patients with p53abn cancers seem to have the highest risk of relapse and death. Molecular subgroups do not appear to predict benefit from VCB/C as compared to pelvic RT. Novel therapeutic approaches are needed, especially for those with dMMR and p53abn expressing cancers.

## 1. Introduction

Endometrial cancer (EC) is the sixth most common malignancy globally with increasing incidence and mortality rates steadily increasing over the past three decades with around 473,614 new cases reported worldwide in 2021 [1]. Although patients with early-stage EC typically have an excellent prognosis, 15–27% of those with high-intermediate risk (defined by combinations of age with grade 2 to 3 histology, lymphovascular space involvement (LVSI), and outer third myometrial invasion) or high-risk disease, experience tumor recurrence [2,3]. Up to 19% of high-risk early-stage patients treated with observation and 10% of those treated with whole pelvic radiation therapy (RT) experience distant recurrence [4,5]. The phase III trial GOG-0249 (NCT00807768) was conducted to determine if the addition of systemic chemotherapy in combination with vaginal brachytherapy (VCB) improved recurrence-free survival (RFS) as compared to pelvic RT. In this trial, Stage I-II EC patients were randomized to either 1) VCB followed by three cycles of carboplatin/paclitaxel chemotherapy (VCB/C) or 2) pelvic RT [3]. Five-year RFS, overall survival (OS), and vaginal and distant recurrence rates were all equivalent between the two arms, though those who received VCB/C had greater acute toxicity and lower quality-of-life. However, this trial did not differentiate patients according to tumor molecular biomarkers, which are now known predictors of outcome [6,7].

Data derived from the Cancer Genome Atlas [8] elucidated molecular risk factors for EC and led to the development of classification algorithms such as ***Pro***active ***Mo***lecular R***is***k Classifier for ***E***ndometrial Cancer (ProMisE) [9] and TransPORTEC [10]. ProMisE uses immunohistochemistry (IHC) to detect deficient mismatch repair (dMMR) and p53 abnormal (p53abn) expressions, and sequencing for polymerase epsilon (*POLE*) exonuclease domain mutations. While the independent prognostic impact of these molecular subgroups is well-established in EC [9,11,12], the value for predicting the benefit of adjuvant therapy in early-stage EC is less certain due to inconsistent findings [6,7]. Most recently, results of PORTEC-4a (NCT03469674), a randomized, open-label phase 3, multicenter non-inferiority trial demonstrated that individualized adjuvant treatment by molecular integrated risk profile is safe and effective for patients with HIR EC [13]. Using molecular-based risk stratification spared 46% of participants with a favorable profile from adjuvant VCB, and reduced both overtreatment and undertreatment. In this era of molecular profiling, there remains a critical need to discover, test, and validate prognostic and predictive biomarkers in EC with the goal of improving outcomes for patients with early-stage disease.

We leveraged a unique opportunity to examine patient samples from GOG-0249 to determine whether a modified ProMisE algorithm using MMR and p53 protein expression predicts RFS and OS benefit in patients with high-risk early-stage I-II EC treated with VCB/C relative to pelvic RT.

## 2. Methods

### 2.1. Participants

Patients were enrolled on GOG-0249 for 46.5 months between 2009 and 2013 [3]. The inclusion criteria for GOG-0249 were described in the original paper. Briefly, this phase III trial (NCT00807768) enrolled 601 patients with International Federation of Gynecology and Obstetrics (2009) stage I endometrioid histology with Gynecologic Oncology Group protocol 33–based high-intermediate–risk criteria [14], stage II disease, or stage I to II serous or clear cell tumors. Six-hundred one eligible patients (12.6% Black, 71.1% White, 16.3% other) were randomly allocated 1:1 to VCB followed by three cycles of paclitaxel 175 mg/m^2^ (3 h) plus carboplatin (AUC 6) every 21 days (VCB/C); or pelvic RT (45 to 50.4 Gy over 5 weeks). Retrospective central pathology review confirmed eligibility. Three hundred one patients were assigned to pelvic RT (*n* = 18 did not receive study treatment) and 300 to VCB/C (*n* = 10 did not receive study treatment). Patients were stratified before randomization by intent to use VCB in the pelvic RT arm and by lymphadenectomy versus no lymphadenectomy. The data cutoff for the initial analysis was December 11, 2016, with a total of 130 events reported for RFS with a median follow-up of 53 months. The same data cutoff was used for this ancillary analysis as there were no further updates on recurrence or survival made after this time. The abstracted patient characteristics included age, race, ethnicity, body mass index, GOG performance status, stage, histology and grade. Clinical trial participation required local or central institutional review board or independent ethics committee approval and all patients signed written informed consent.

This ancillary analysis was proposed and approved through the National Clinical Trials Network (NCTN) Navigator NCTN Core Correlative Sciences Committee. This study was conducted under the “Biomarker Assessment in Gynecologic Cancer” protocol that was approved by the lead institution's institutional review board on December 12, 2022. This study adhered to the REMARK (Reporting Recommendations for Tumor Marker Prognostic Studies) guidelines.

### 2.2. Specimens and laboratory testing

Formalin-fixed, paraffin-embedded (FFPE) tumor tissue blocks or unstained slides from 323 patients enrolled on GOG-0249 and stored at the GOG/NRG Oncology Biospecimen Bank-Columbus were available for analysis. Eight slides had incomplete molecular data to complete classification and ultimately these patients were removed from our analysis. Unstained FFPE tumor tissue sections mounted on glass slides were shipped from sites at room temperature and either stored at room temperature or wax-dipped and stored at 4 °C in vacuum-sealed pouches upon receipt at the biobank; they were subsequently removed from storage to be processed for this study. The FFPE tumor tissue blocks submitted were shipped from sites at room temperature and subsequently banked at 4 °C upon receipt at the biobank until 2022; at that time, they were moved to room temperature storage until processed for this study.

Tumor samples were analyzed using IHC to assess protein expression for MMR and p53. IHC was performed at Duke University Health System Clinical Laboratories which is accredited by the College of American Pathologists and certified under CLIA standards. Staining was performed using primary antibodies for MLH1 (G168–728) (1:100), MSH2 (G2019–1129)(1:1000), PMS2 (MRQ-28) (1:100), MSH6 (44) (1:200) (Cell Marque, Sigma-Aldrich, St. Louis, MO), and p53 (DO7)(Leica biosystems, Buffalo Grove, IL) which were incubated overnight at 4 °C. Slides were then rinsed with phosphate-buffered saline, incubated for one hour at room temperature with a horseradish peroxidase–conjugated secondary antibody in blocking buffer, developed with 3, 3″-diaminobenzidine chromogen (Invitrogen, Carlsbad, CA), and then counterstained with Gil's No. 3 hematoxylin (Sigma-Aldrich, St. Louis, MO). FFPE cut sections containing non-neoplastic tissue served as appropriate internal positive and negative controls. All stained slides were evaluated by a board-certified pathologist with expertise in cytopathology and women's and perinatal pathology (KS), who was blinded to patient clinicopathologic data, treatment group, and survival outcomes.

### 2.3. Molecular classification

A modified ProMisE algorithm, previously described by Clements et a l. [15] for EC was used to molecularly classify the tumors for this analysis. Briefly, the modified algorithm used IHC for the four MMR proteins (MLH1, PMS2, MSH6 and MSH2) and p53. Tumors were first designated based on MMR status and classified as either deficient (dMMR) or proficient. If proficient, the tumor was further classified based on p53 status—wildtype (p53wt) or abnormal (p53abn). Consistent with prior work published on multiple classifiers [16], tumors that were dMMR and p53abn were classified as dMMR.

### 2.4. Statistical analysis

The primary end point for this study was RFS, defined as time from randomization to date of first relapse or death, whichever occurred first. Patients alive without recurrence were censored on the date of their last scan. Due to the small number of events, descriptive statistics were used to describe the incidence and pattern of recurrences. The secondary endpoint was OS, defined as the time from randomization to date of death of any cause. Alive patients were censored on the date of last contact. Clinicopathological characteristics within molecular subgroups were compared with a chi-squared test for categorical variables or the Wilcoxon rank sum test for continuous variables. Clinicopathological characteristics of patients included in this study and the complete GOG-0249 cohort were descriptively compared.

Multivariable proportional hazards models were used to estimate the (log) hazard ratios (HRs) and their variances for molecular subgroups for both RFS and OS separately. Each model included assigned treatment and ProMisE subgroup indicators. For the predictive analysis, multivariable proportional hazards models were used to estimate the (log) treatment HRs (VCB/C relative to RT) and their variances by molecular subgroup for both RFS and OS separately. Each model included assigned treatment, ProMisE subgroup indicators, and an interaction between treatment and ProMisE subgroup indicators. All models described included stratification factors used in the primary analysis: nodal surgery stratum and planned use of VCB. Unadjusted Kaplan-Meier estimates of RFS and OS distributions by treatment and molecular subgroup were plotted and used to estimate 5-year survival rates. Median follow-up was estimated by use of reverse Kaplan Meier method. *P*-values less than 0.05 were considered statistically significant. Statistical analysis was performed with SAS software, version 9.4.

## 3. Results

### 3.1. Patient characteristics

**Fig. 1.**
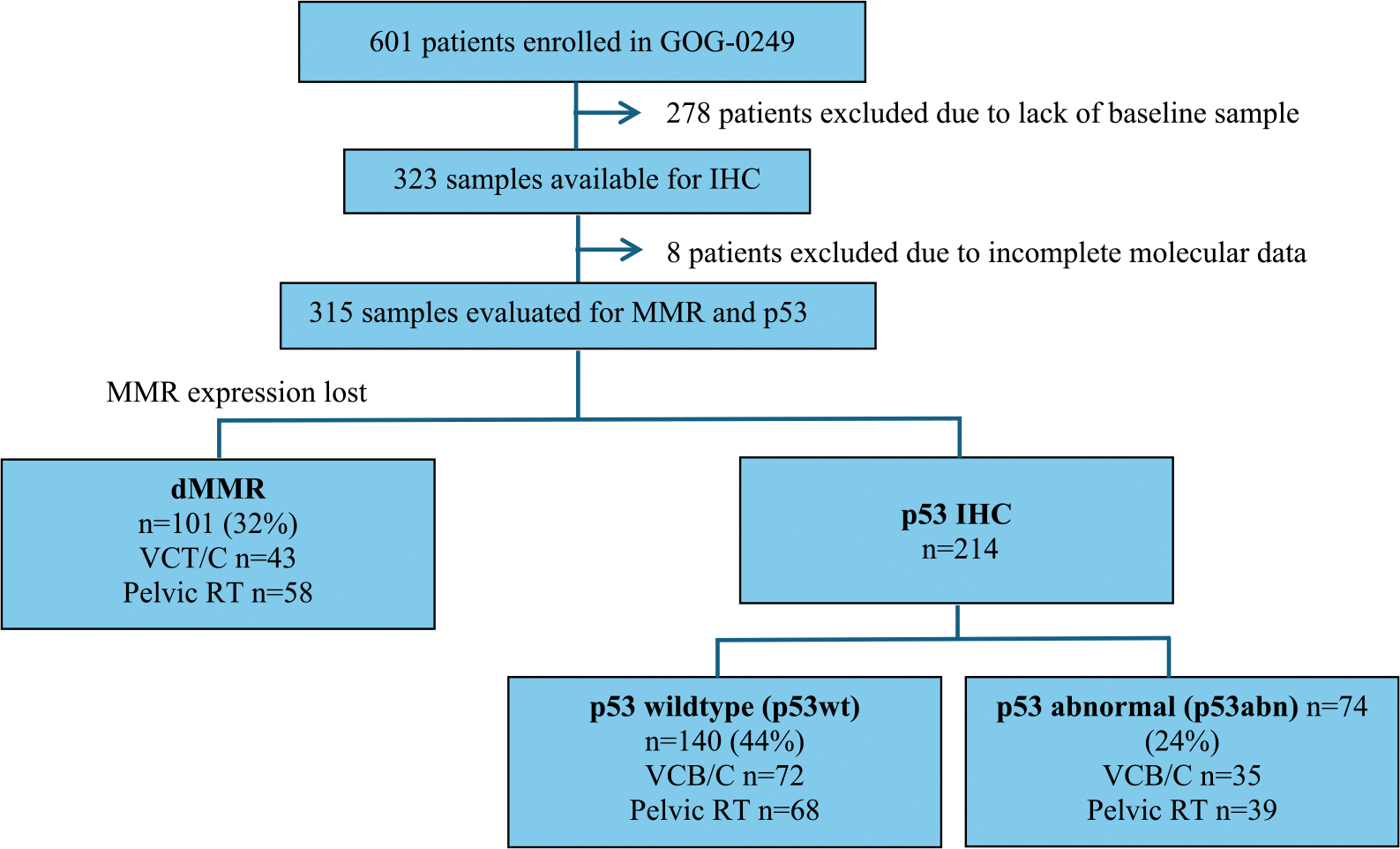
Flow chart of immunohistochemistry staining. IHC = Immunohistochemistry; MMR = Mismatch repair; dMMR = Mismatch repair deficient; VCB/C = Vaginal cuff brachytherapy followed by three cycles of carboplatin/paclitaxel chemotherapy; RT = Radiation therapy.

Of the 601 patients who enrolled on the parent protocol, 315 (52.4%) patients had complete survival data and IHC staining of baseline tumor samples and were included in this ancillary analysis (Fig. 1). The patient and tumor characteristics for the ancillary subgroup appeared similar to the trial intent-to-treat population overall (Supplementary Table 1). Median follow-up was 54 months (range: 0.1 months to 88.1 months).

Using a modified ProMisE algorithm, 32.1% were classified as dMMR, 23.5% of ECs p53abn, and 44.4% p53wt. Associations between staining patterns (p53abn, dMMR, p53wt) and clinicopathologic variables are summarized in Table 1. Notably, p53abn ECs were more frequent in patients who were older (*p* = 0.004), diagnosed with serous histology (*p* < 0.001), underwent lymphadenectomy (*p* = 0.02) and received planned VCB (p < 0.001). There were no differences in race, ethnicity, stage, performance status, and body mass index between molecular subgroups.

### 3.2. Survival outcomes

**Fig. 2.**
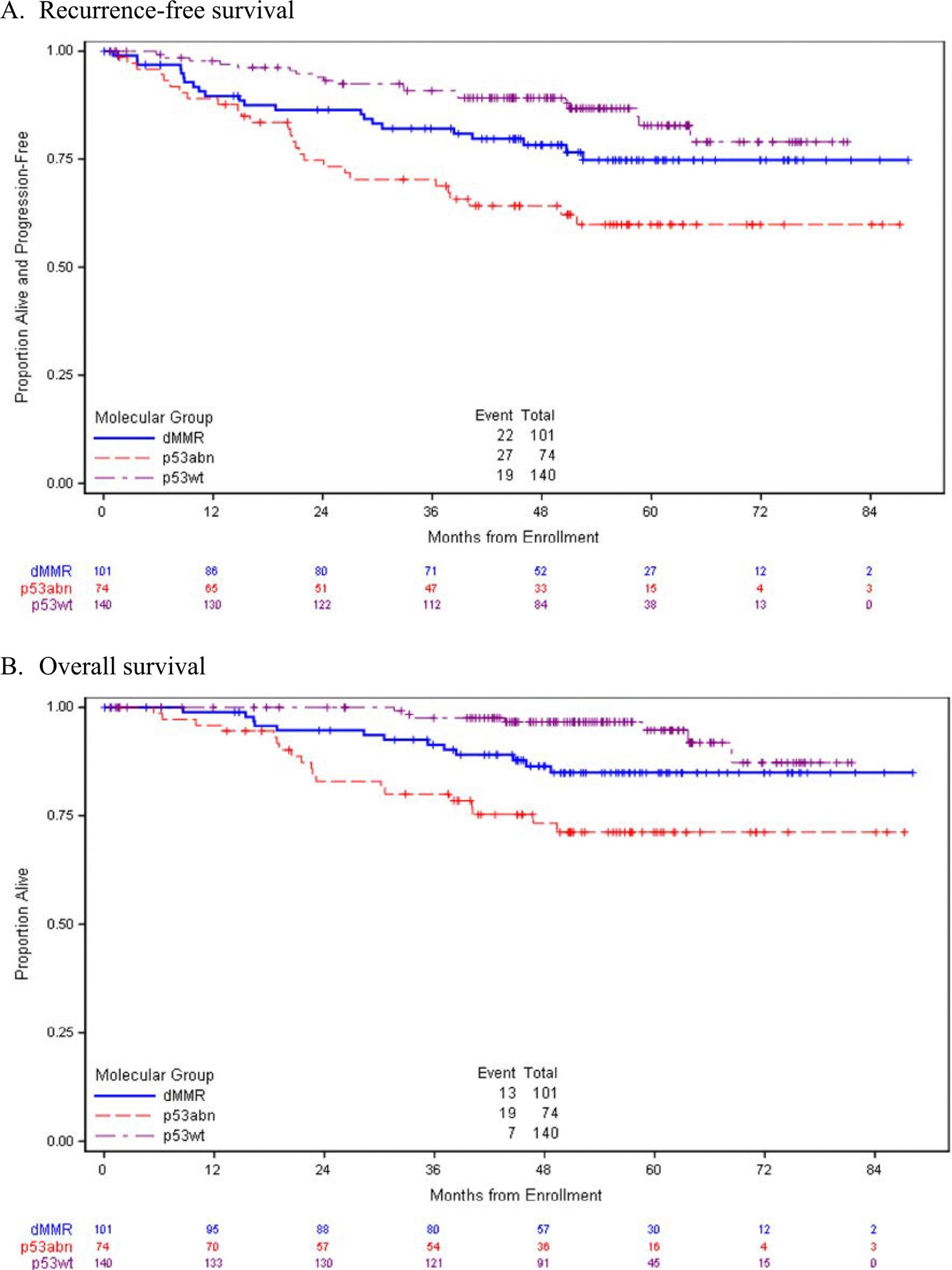
Five-year recurrence-free and overall survival by molecular subgroup. dMMR = Mismatch repair deficient; p53abn = p53 abnormal; p53wt = p53 wildtype.

Molecular classification using the modified ProMisE algorithm was prognostic for RFS and OS (Fig. 2). Five-year RFS and OS were worse in patients with p53abn cancers, 58.7% [HR = 4.0 (95% Confidence Interval (CI): 2.1–7.5)] and 70.7% [HR = 9.4 (95%CI: 3.8–23.6)] and dMMR cancers were 74.4% [HR = 1.8 (95%CI: 0.9–3.3)] and 84.3% [HR = 3.0 (95% CI: 1.2–7.8)] compared to those with p53wt (referent) (83.4% and 95.3%)(p < 0.001) (Table 2).

The differences in adjuvant treatment effect (VCB/C vs pelvic RT) among the molecular subgroups by five-year RFS (Supplementary Fig. 1) and OS (Supplementary Fig. 2) were analyzed. The test for interaction between molecular subgroups and treatment arm did not reach significance (RFS: *p* = 0.7; OS: *p* = 0.8). However, we were still interested in the directions of the treatment HRs within each molecular subgroups, which we could not gleam from the interaction test, and thus proceeded with the following exploratory analyses. Although patients with p53abn EC treated with VCB/C numerically had a higher five-year RFS rate than those treated with RT [RFS: 65.1% vs 52.0%; HR = 0.7 (0.3–1.5)], these findings did not reach statistical significance. The five-year OS rates for patients with p53abn cancers were 68.3% for those treated with VCB/C and 73.8% for with pelvic RT [HR = 0.99 (0.4–2.5)]. Five-year RFS and OS outcomes were similar for individuals with dMMR [RFS: 74.4% vs 74.3%, HR = 1.2 (0.5–2.7); OS: 83.5% vs 85.0%, HR = 1.6 (0.5–5.0)] and p53wt cancers [RFS: 84.0% vs 82.7%, HR = 0.7 (0.3–1.7); OS: 94.1% vs 96.5%, HR = 1.2 (0.3–5.6)].

### 3.3. Recurrence patterns

Overall, 18.7% (59/315) of patients experienced a recurrence, which is consistent with the number of recurrences reported in the original GOG-0249 trial [3]. Of these 59 recurrences, 3 (5%) were vaginal, 24 (41%) pelvic and/or para-aortic, and 32 (54%) distant. Patients treated with VCB/C had higher numerical rates of pelvic recurrences, while those treated with pelvic RT had higher rates of distant metastasis, regardless of molecular subtype (Table 3). When assessed by molecular subgroup, the distant recurrences were two times more likely in patients with p53abn EC compared to those with dMMR or p53wt cancers. Pelvic or para-aortic nodal recurrences were most common in the p53abn group.

## 4. Discussion

Our post-hoc, ancillary study demonstrates that molecular characterization using a modified ProMisE algorithm has prognostic value in patients with high-intermediate and high-risk, early-stage EC who were enrolled in GOG-0249. Because of the randomized design of GOG-0249, it is the first, to our knowledge, to explore the potential predictive value of molecular classification of EC for response to VCB/C versus pelvic RT. There were no differences in survival outcomes when comparing VCB/C to pelvic RT by molecular subgroup. While the five-year RFS rates were higher in patients with p53abn cancers treated with VCB/C compared to pelvic RT, this finding was not statistically significant and did not lead to better OS. Notably, the addition of chemotherapy to VCB did not appear to significantly decrease relapse or improve survival in patients with dMMR or p53wt ECs. The survival outcomes were strikingly similar regardless of treatment modality. Thus, suggesting that the side effects, extended treatment, and costs of chemotherapy may be avoided in these patient groups.

Although pelvic RT was the control arm in GOG-0249, its consistent lack of OS benefit in early-stage EC underscores the need to better understand which groups benefit the most from this adjuvant therapy. Current National Comprehensive Cancer Network guidelines recommend external beam RT for Stage 1B grade 3 with or without systemic therapy [17]. For stage II grade 1–3, external beam RT is preferred over VCB, with or without systemic therapy. Real world use of VCB/C among women meeting GOG-0249 criteria was evaluated in 7548 women identified in the National Cancer Database (NCDB). Chodavadia et al. [18] reported that VCB/C receipt from 2004 to 2015 was associated with higher socioeconomic status (*p* < 0.001), higher grade endometrioid cancer (p < 0.001), and aggressive histology (p < 0.001). After propensity-score matching, and stratification by FIGO stage, VCB/C was associated with improved OS for only FIGO stage 1B (HR 0.62, 95% CI 0.44–0.87). However, these findings and others in the literature are limited by potential selection bias and underpowered post hoc analyses. Moreover, molecular classification was not included in this analysis, which is a notable limitation of the NCDB study, but underscores important opportunities to incorporate molecular tumor markers into large databases for future research.

In the pooled molecular analysis of the randomized PORTEC-1 and PORTEC-2 trials for early-stage EC, there was a substantial RFS benefit among early-stage patients with p53abn EC (*n* = 70) who received adjuvant external beam RT (96.9%) compared to VCB (64.3%) and no adjuvant therapy (72.2%), *p* = 0.048 [6]. While our results reflect comparable rates of five-year RFS for the experimental arm of GOG-0249, VCB/C (65.1% vs 64.3%), we acknowledge our lower rates achieved with pelvic RT (52% vs 96.9%), may be related to differences in eligibility criteria. GOG-0249 included “higher-risk” patients compared to the PORTEC-1 and -2 trials, including 32 (43.2%) vs 14 (1.6%) patients with serous histology (PORTEC-2 excluded serous histology), and stage II disease (14, 18.9% vs 2, 0.2%). Furthermore, while lymphadenectomy was not a requirement of PORTEC-1, PORTEC-2 or GOG-0249, 95% of patients included in our ancillary analysis with p53abn EC were surgically staged.

We can also glean insights into this high-risk group from more recently published studies and ongoing trials. The molecular analysis of the PORTEC-3 trial for high-risk EC showed a benefit of added chemotherapy observed in all patients with p53abn EC across all stages (RFS: HR 0.52, *p* = 0.022) partly corresponding to the benefit seen in serous EC [7]. However, in their exploratory subanalyses, the number of patients with Stage I/II and p53abn EC were small (*n* = 61; RFS: 26 events; OS: 23 events), limiting their power to detect whether molecular classification predicts response to adjuvant therapy. Moreover, the findings may be due to multiplicity and risk of false positive results. Nevertheless, consistent with our study findings, they showed that a suggested five-year RFS benefit of chemotherapy combined with RT in the p53abn group [75.0% vs 44.6%, HR 0.40 (0.18–0.90), P_cox_ = 0.027] did not translate into a five-year OS benefit [74.6% vs 49.6%, HR 0.47 (0.20–1.20), P_cox_ = 0.082]. Taken together with other ancillary studies of molecular classification of randomized trials involving early-stage EC [6], our collective data underscores an urgent need for novel therapeutic interventions to reduce recurrence rates and improve survival outcomes in patients with dMMR and p53abn EC.

The landscape of EC care continues to evolve rapidly. The recent ENGOT en-11/GOG-3053/KEYNOTE-B21 randomized, double-blind, phase II study of pembrolizumab or placebo plus adjuvant chemotherapy with or without RT included select patients with high-risk stage I or II disease and those with surgical stage III or IVA disease of any histology [19]. While they did not show improved disease-free survival with adjuvant pembrolizumab plus chemotherapy in all comers, their pre-planned subgroup analyses suggested that there was benefit in patients with dMMR EC. PORTEC-4a (NCT03469674) was a randomized, open-label phase 3, multicenter non-inferiority trial that investigated molecular risk profile-based individualized adjuvant therapy for women with HIR EC [13]. Patients were randomized 2:1 to either adjuvant treatment according to their molecular integrated risk profile or to standard VCB. In the molecular group, 168 (46%) had a favorable profile (*POLE*-mutated or NSMP-*CTNNB1* wildtype) and were observed; 148 (40%) had an intermediate profile (dMMR or NSMP-*CTNNB1* wildtype) and received VCB; and 51 (14%) had an unfavorable profile (p53abn or substantial LVSI or L1 cell adhesions molecule overexpression) and received pelvic RT. The primary endpoint, 5-year cumulative incidence of vaginal recurrence was 4.5% (95% CI 2.23–6.76) in the molecular profile group and 1.6% (0.00–3.32) in the standard group (HR 2.71 [95% CI 0.79–9.34]). Individualized adjuvant treatment by molecular risk spared 46% of patients with a favorable profile from adjuvant treatment and reduced both overtreatment and undertreatment.

The GY020 (NCT04214067) is a phase III trial that will determine if the addition of pembrolizumab to RT in patients with dMMR early-stage HIR EC is more effective than RT alone to improve RFS. Several ongoing trials, such as the GY026 (NCT05256225) and GY032 (NCT06388018) are exploring integral molecular biomarkers to either direct targeted therapy or adjuvant RT. These pivotal studies are laying the foundation for innovative biomarker-specific randomized controlled trials and driving a new era of precision-based medicine to improve outcomes for patients with EC and offset the rising mortality.

Given the inherent limitations of this ancillary analysis, our study was not powered to detect a difference between treatment arms within molecular subgroups. With the sample size, our power ranged between 5% and 37% for RFS and 8% and 20% for OS. Specifically, in the p53abn group, there was 37% power for RFS and 15% for OS. We also acknowledge the abbreviated nature of our modified ProMisE algorithm. *POLE* analysis in GOG-0249 patient samples is currently ongoing and not included in this analysis. Nevertheless, it is well established that *POLE* mutations, even in high-risk, advanced-stage EC and non-endometrioid histologies confer excellent survival, and we expect the prevalence of *POLE* mutations to be less than 10% [20]. In addition, our analysis is limited to 52.4% of the overall GOG-0249 study population due to the availability of archival tissue matched with survival data. Although the demographics and pathologic characteristics of this molecular cohort are similar to the overall study population (Supplemental Table 1), we acknowledge the limited representation of racial and ethnic minoritized patients despite enrollment across 63 sites in the United States. Lastly, use of VCB boost was not standardized in the RT arm. Per protocol, it was permitted for patients with cervical involvement or serous or clear cell histology and received by 126 out of 145 (86.9%) eligible patients, with an increased bias for use in patients with p53abn EC.

In conclusion, molecular classification is prognostic for survival in patients with HIR/high-risk stage I-II EC, but does not appear to predict the benefit of VCB/C relative to pelvic RT. The findings suggest that molecular classifiers may be useful to provide more informative patient counseling regarding recurrence risk and survival outcomes. Further research is urgently needed to identify novel adjuvant therapies based on molecular classification, especially those targeting *TP53* alterations and defects in MMR protein expression. As we advance our risk stratification beyond those histopathologic features defined in GOG-33, to now include molecular classifiers, we can use ancillary data to better inform the design of future clinical trials. Opportunities to drive research forward also include the strategic combination of ancillary datasets and continued support for correlative biospecimen studies. Excitingly, advances in molecular profiling of EC, alongside the introduction of novel drugs, are transforming the landscape of precision medicine in EC.

## Supplementary Material

MMC4

MMC3

MMC2

MMC1

## Figures and Tables

**Table 1:** Baseline patient and tumor characteristics by molecular status.

Characteristic	dMMR*	p53wt	p53abn	P
	(*n* = 101)	(*n* = 140)	(*n* = 74)	

Median age (range), y	63 (39–80)	61.5 (25–89)	64.5 (50–86)	0.004
	Race n (%)			
Asian	3 (3.0)	8 (5.7)	4 (5.4)	0.07
Black	8 (7.9)	13 (9.3)	17 (23.0)	
White	83 (82.2)	113 (80.7)	47 (63.5)	
Other	3 (3.0)	2 (1.4)	3 (4.1)	
Not specified	4 (4.0)	4 (2.9)	3 (4.1)	
	Ethnicity			
Hispanic or Latinx	7 (6.9)	6 (4.3)	7 (9.5)	0.39
Non-Hispanic	90 (89.1)	131 (93.6)	63 (85.4)	
Not specified	4 (4.0)	3 (2.1)	4 (5.4)	
	FIGO Stage			
I	75 (74.3)	98 (70.0)	60 (81.1)	0.21
II	26 (25.7)	42 (30.0)	14 (18.9)	
	Histology			
Endometrioid, Grade 1	14 (13.9)	42 (30.0)	2 (2.7)	<0.001
Endometrioid, Grade 2	47 (46.5)	61 (43.6)	8 (10.8)	
Endometrioid, Grade 3	29 (28.7)	19 (13.6)	20 (27.0)	
Endometrioid, not graded	0 (0.0)	0 (0.0)	1 (1.4)	
Serous	5 (5.0)	7 (5.0)	32 (43.2)	
Clear cell	1 (1.0)	5 (3.6)	3 (4.1)	
Mixed epithelial	5 (5.0)	5 (3.6)	6 (8.1)	
Undifferentiated	0 (0.0)	0 (0.0)	1 (1.4)	
Adenocarcinoma NOS	0 (0.0)	0 (0.0)	1 (1.4)	
Other	0 (0.0)	1 (0.7)	0 (0.0)	
	Performance status			
0	75 (74.3)	106 (75.7)	54 (73.0)	0.19
1	22 (21.8)	34 (24.3)	19 (25.7)	
2	4 (4.0)	0 (0.0)	1 (1.4)	
	Body Mass Index category			
Normal or Underweight	14 (13.9)	22 (15.7)	10 (13.5)	0.27
Overweight	29 (28.7)	21 (15.0)	19 (25.7)	
Obese Class I (Moderate)	20 (19.8)	38 (27.1)	16 (21.6)	
Obese Class II (Severe)	15 (14.9)	19 (13.6)	14 (18.9)	
Obese Class III (Very Severe)	23 (22.8)	40 (28.6)	15 (20.3)	
	Nodal surgery stratum			
No lymphadenectomy	8 (7.9)	23 (16.4)	4 (5.4)	0.024
Lymphadenectomy	93 (92.1)	117 (83.6)	70 (94.6)	
	Planned use of VCB			
Not allowed	61 (60.4)	87 (62.1)	22 (29.7)	<0.001
Optional but not planned	4 (4.0)	8 (5.7)	7 (9.5)	
Optional and planned	36 (35.6)	45 (32.1)	45 (60.8)	

dMMR = mismatch repair deficient; p53wt = p53 wildtype; p53abn = p53 abnormal; NOS = Not otherwise specified; VCB = Vaginal cuff brachytherapy.

* There were three patients who were both dMMR and p53abn, that were classified as dMMR.

**Table 2:** Recurrence-free and overall survival and adjusted hazard ratios based on molecular subgroup.

	5-year RFS rate (%)	p-value*	HR (95% CI) *	5-year OS rate (%)	p-value*	HR (95% CI)*

dMMR	74.4	<0.001	1.76 (0.93–3.31)	84.3	<0.001	3.01 (1.16–7.81)
p53abn	58.7		3.97 (2.11–7.50)	70.7		9.41 (3.75–23.60)
p53wt	83.4		Reference	95.3		Reference
dMMR VCB/C vs RT	74.4 vs 74.3	0.66	1.15 (0.48–2.74)	83.5 vs 85.0	0.82	1.60 (0.51–5.01)
p53abn VCB/C vs RT	65.1 vs 52.0		0.71 (0.33–1.52)	68.3 vs 73.8		0.99 (0.39–2.54)
p53wt VCB/C vs RT	84.0 vs 82.7		0.69 (0.28–1.73)	94.1 vs 96.5		1.25 (0.28–5.61)

dMMR = Mismatch repair deficient; p53abn = p53 abnormal; p53wt = p53 wildtype; VCB/C = Vaginal cuff brachytherapy followed by three cycles of carboplatin/paclitaxel chemotherapy; RT = Radiation therapy; RFS = Recurrence-free survival; HR = Hazard radio; OS = Overall survival.

* Adjusted for nodal surgery stratum, planned use of VCB stratum, and treatment.

**Table 3:** Primary sites of recurrence by molecular subgroup and treatment.

Primary site of recurrence, n (%)	dMMR		p53wt		P53abn	
	RT	VCB/C	RT	VCB/C	RT	VCB/C

Vagina	1 (11.1)	1 (12.5)	0 (0.0)	1 (14.3)	0 (0.0)	0 (0.0)
Pelvis	2 (22.2)	3 (37.5)	1 (10.0)	4 (57.1)	1 (8.3)	5 (38.5)
Para-aortic	1 (11.1)	2 (25.0)	2 (20.0)	0 (0.0)	1 (8.3)	0 (0.0)
Pelvis + Para-aortic	1 (11.1)	0 (0.0)	0 (0.0)	0 (0.0)	0 (0.0)	1 (7.7)
Distant	4 (44.4)	2 (25.0)	7 (70.0)	2 (28.6)	10 (83.3)	7 (53.9)
Total	9 (100.0)	8 (100.0)	10 (100.0)	7 (100.0)	12 (100.0)	13 (100.0)

dMMR = Mismatch repair deficient; p53wt = p53 wildtype; p53abn = p53 abnormal; RT = Radiation therapy; VCB/C = Vaginal cuff brachytherapy followed by three cycles of carboplatin/paclitaxel chemotherapy.

## HIGHLIGHTS

- Molecular classification using a modified ProMisE algorithm appears prognostic in early-stage endometrial cancer.
- Patients with p53 abnormal expressing endometrial cancers have the highest risk of relapse and death.
- Molecular classifiers do not appear to predict benefit from chemotherapy and brachytherapy relative to pelvic radiation.

## Appendix A. Supplementary data

Supplementary data to this article can be found online at https://doi.org/10.1016/j.ygyno.2026.05.013.

## References

[1] Zhu X, , Global burden of uterine cancer in 204 countries and territories and its predicted level in 15 years, from 1990 to 2021, J. Gynecol. Oncol. 36 (6) (2025), e125. 40537983 10.3802/jgo.2025.36.e125 PMC12636118

[2] Keys HM, , A phase III trial of surgery with or without adjunctive external pelvic radiation therapy in intermediate risk endometrial adenocarcinoma: a gynecologic oncology group study, Gynecol. Oncol. 92 (3) (2004) 744–751. 14984936 10.1016/j.ygyno.2003.11.048

[3] Randall ME, , Phase III trial: adjuvant pelvic radiation therapy versus vaginal brachytherapy plus paclitaxel/carboplatin in high-intermediate and high-risk early stage endometrial cancer, J. Clin. Oncol. 37 (21) (2019) 1810–1818. 30995174 10.1200/JCO.18.01575 PMC6804858

[4] Creutzberg CL, , Fifteen-year radiotherapy outcomes of the randomized PORTEC-1 trial for endometrial carcinoma, Int. J. Radiat. Oncol. Biol. Phys. 81 (4) (2011) e631–e638. 21640520 10.1016/j.ijrobp.2011.04.013

[5] Nout RA, , Long-term outcome and quality of life of patients with endometrial carcinoma treated with or without pelvic radiotherapy in the post operative radiation therapy in endometrial carcinoma 1 (PORTEC-1) trial, J. Clin. Oncol. 29 (13) (2011) 1692–1700. 21444867 10.1200/JCO.2010.32.4590

[6] Horeweg N, , Molecular classification predicts response to radiotherapy in the randomized PORTEC-1 and PORTEC-2 trials for early-stage endometrioid endometrial cancer, J. Clin. Oncol. 41 (27) (2023) 4369–4380. 37487144 10.1200/JCO.23.00062 PMC10522107

[7] Leon-Castillo A, , Molecular classification of the PORTEC-3 trial for high-risk endometrial cancer: impact on prognosis and benefit from adjuvant therapy, J. Clin. Oncol. 38 (29) (2020) 3388–3397. 32749941 10.1200/JCO.20.00549 PMC7527156

[8] Cancer Genome Atlas Research, N, , Integrated genomic characterization of endometrial carcinoma, Nature 497 (7447) (2013) 67–73. 23636398 10.1038/nature12113 PMC3704730

[9] Talhouk A, , Confirmation of ProMisE: a simple, genomics-based clinical classifier for endometrial cancer, Cancer 123 (5) (2017) 802–813. 28061006 10.1002/cncr.30496

[10] Stelloo E, , Refining prognosis and identifying targetable pathways for high-risk endometrial cancer; a TransPORTEC initiative, Mod. Pathol. 28 (6) (2015) 836–844. 25720322 10.1038/modpathol.2015.43

[11] Stelloo E, , Improved risk assessment by integrating molecular and clinicopathological factors in early-stage endometrial cancer-combined analysis of the PORTEC cohorts, Clin. Cancer Res. 22 (16) (2016) 4215–4224. 27006490 10.1158/1078-0432.CCR-15-2878

[12] Kommoss S, , Final validation of the ProMisE molecular classifier for endometrial carcinoma in a large population-based case series, Ann. Oncol. 29 (5) (2018) 1180–1188. 29432521 10.1093/annonc/mdy058

[13] van den Heerik A, , Molecular profile-based adjuvant treatment for women with high-intermediate risk endometrial cancer (PORTEC-4a): results of a randomised, open-label, phase 3, multicentre, non-inferiority trial, Lancet Oncol. 27 (1) (2026) 23–35. 41449145 10.1016/S1470-2045(25)00612-6

[14] Creasman WT, , Surgical pathologic spread patterns of endometrial cancer. A gynecologic oncology group study, Cancer 60 (8 Suppl) (1987) 2035–2041. 3652025 10.1002/1097-0142(19901015)60:8+<2035::aid-cncr2820601515>3.0.co;2-8

[15] Clements A, , Molecular classification of endometrial cancers (EC) and association with relapse-free survival (RFS) and overall survival (OS) outcomes: ancillary analysis of GOG-0258, Gynecol. Oncol. 193 (2025) 119–129. 39854806 10.1016/j.ygyno.2025.01.006 PMC11929956

[16] Leon-Castillo A, , Clinicopathological and molecular characterisation of ‘multiple-classifier’ endometrial carcinomas, J. Pathol. 250 (3) (2020) 312–32 2. 31829447 10.1002/path.5373 PMC7065184

[17] National Comprehensive Cancer Network Clinical Practice Guidelines in Oncology Uterine Neoplasms Version 3.2025, https://www.nccn.org/guidelines/category_1 2025 Last accessed March 7, 2026.

[18] Chodavadia PA, , Off-study utilization of experimental therapies: analysis of GOG249-eligible cohorts using real world data, Gynecol. Oncol. 156 (1) (2020) 154–161. 31759772 10.1016/j.ygyno.2019.09.017 PMC8397368

[19] Van Gorp T, , ENGOT-en11/GOG-3053/KEYNOTE-B21: a randomised, double-blind, phase III study of pembrolizumab or placebo plus adjuvant chemotherapy with or without radiotherapy in patients with newly diagnosed, high-risk endometrial cancer, Ann. Oncol. 35 (11) (2024) 968–980. 39284383 10.1016/j.annonc.2024.08.2242

[20] McConechy MK, , Endometrial carcinomas with POLE exonuclease domain mutations have a favorable prognosis, Clin. Cancer Res. 22 (12) (2016) 2865–2873. 26763250 10.1158/1078-0432.CCR-15-2233

