# Supplementary material for: Molecular classification and association with survival outcomes in high-intermediate and high-risk early-stage endometrial cancers: Ancillary analysis of GOG-0249
Source: Gynecol Oncol. Author manuscript; Available in PMC 2026 Jul 20. (PMC13382316; doi:10.1016/j.ygyno.2026.05.013)
Supplement: MMC3 [file NIHMS2188080-supplement-MMC3.docx]

Supplementary Table 1. Baseline Patient and Tumor Characteristics in Eligible GOG-0249 Patients versus

Patients included in Modified ProMisE Algorithm Analysis

| Characteristic | GOG-0249  Patients  (n=601) | Modified ProMiSe Patients  (n=315) |
| --- | --- | --- |
| Median age (range), y | 62 (25-89) | 62 (25-89) |
| Race n (%) |  |  |
| Asian | 50 (8.3) | 15 (4.8) |
| Black | 80 (13.3) | 38 (12.1) |
| White | 432 (71.9) | 243 (77.1) |
| Other | 15 (2.5) | 8 (2.5) |
| Not specified | 24 (4.0) | 11 (3.5) |
| Ethnicity |  |  |
| Hispanic or Latinx | 42 (7.0) | 20 (6.4) |
| Non-Hispanic | 523 (87.0) | 284 (90.2) |
| Not specified | 36 (6.0) | 11 (3.5) |
| FIGO Stage |  |  |
| I | 451 (75.0) | 233 (74.0) |
| II | 149 (24.8) | 82 (26.0) |
| IV | 1 (0.2) | 0 (0.0) |
| Histology |  |  |
| Endometrioid, Grade 1 | 106 (17.6) | 58 (18.4) |
| Endometrioid, Grade 2 | 212 (35.3) | 116 (36.8) |
| Endometrioid, Grade 3 | 125 (20.8) | 68 (21.6) |
| Endometrioid, not graded | 1 (0.2) | 1 (0.3) |
| Serous | 88 (14.6) | 44 (14.0) |
| Clear cell | 28 (4.7) | 9 (2.9) |
| Mixed epithelial | 31 (5.2) | 16 (5.1) |
| Undifferentiated | 3 (0.5) | 1 (0.3) |
| Adenocarcinoma NOS | 2 (0.3) | 1 (0.3) |
| Other | 4 (0.7) | 0 (0.0) |
| Performance status |  |  |
| 0 | 463 (77.0) | 235 (74.6) |
| 1 | 130 (21.6) | 75 (23.8) |
| 2 | 8 (1.3) | 5 (1.6) |
| BMI category |  |  |
| Normal or Underweight | 111 (18.4) | 46 (14.6) |
| Overweight | 136 (22.6) | 69 (21.9) |
| Obese Class I (Moderate) | 130 (21.6) | 74 (23.5) |
| Obese Class II (Severe) | 91 (15.1) | 48 (15.2) |
| Obese Class III (Very Severe) | 133 (22.1) | 78 (24.8) |
| Nodal surgery stratum |  |  |
| No lymphadenectomy | 64 (10.6) | 35 (11.1) |
| Lymphadenectomy | 537 (89.4) | 280 (88.9) |
| Planned use of VCB |  |  |
| Not allowed | 312 (51.9) | 170 (54.0) |
| Optional but not planned | 47 (7.8) | 19 (6.0) |
| Optional and planned | 242 (40.3) | 126 (40.0) |

*There were three patients who were both dMMR and p53abn, that were classified as dMMR.

FIGO = International Federation of Gynecology and Obstetrics; NOS=Not otherwise specified; BMI = body mass index; VCB= Vaginal cuff brachytherapy; ProMisE ***=*** Proactive Molecular Risk Classifier for Endometrial Cancer
