# Supplementary material for: Molecular classification and association with survival outcomes in high-intermediate and high-risk early-stage endometrial cancers: Ancillary analysis of GOG-0249
Source: Gynecol Oncol. Author manuscript; Available in PMC 2026 Jul 20. (PMC13382316; doi:10.1016/j.ygyno.2026.05.013)
Supplement: MMC2 [file NIHMS2188080-supplement-MMC2.docx]

Supplementary Figure 2: Intention-to-treat analysis of 5-year overall survival based on molecular subgroups.

1. dMMR

(B) p53abn

(C) p53wt

dMMR = Mismatch repair deficient; p53abn = p53 abnormal; p53wt = p53 wildtype; RT = Radiation therapy; VCB/C = Vaginal cuff brachytherapy followed by three cycles of carboplatin/paclitaxel chemotherapy
